# The Significance of ST Depression in a Postmenopausal Woman on Estrogen Therapy during Regadenoson Myocardial SPECT Imaging

**DOI:** 10.1155/2015/653760

**Published:** 2015-04-15

**Authors:** Nishaki Kiran Mehta, Charles Hardebeck, Martha Gulati

**Affiliations:** Division of Cardiovascular Medicine, The Ohio State University Wexner Medical Center, 473 West 12th Avenue, Suite 200, Columbus, OH 43210, USA

## Abstract

The incidence of false-positive stress tests has been noted in women, especially on hormone replacement therapy. Current literature describes this phenomenon in treadmill and adenosine stress tests. The introduction of regadenoson as a vasodilator agent has been widely adopted owing to its potency and specificity. To our knowledge, false-positive stress test with regadenoson in a postmenopausal woman on estrogen has never been described. Given the higher chronotropic response with regadenoson, we believe that normal perfusion images with a higher heart rate response indicate a good prognosis in such patients.

## 1. Introduction

Whereas mild-to-moderate ST changes in the presence of normal single photon emission computed tomography (SPECT) images (+EKG/−SPECT) during exercise is generally considered a “false-positive” EKG response and thus a low-risk pattern, the diagnostic and prognostic significance of this combination during vasodilator stress is more controversial [1–8]. While +EKG/−SPECT during vasodilator stress likely represents benign (nonischemic) finding in many patients, elevated rates of cardiac events or revascularization have been noted in association with elevated baseline risk (established coronary disease, diabetics, and elderly patients) [3, 4, 7]; recent commentaries have thus recommended that decisions regarding additional testing in this circumstance consider the full clinical context leading to the exam [2, 6].

Published analyses of +EKG/−SPECT with vasodilator stress note a predominance in female patients (77–88%) of postmenopausal age (mean age 65–72) [3–5, 7, 8]. Although these studies excluded certain patients likely to have stress-induced ST changes unassociated with coronary disease (LBBB, resting ST changes, and digoxin therapy), possible association with estrogen replacement therapy (ERT) was generally not considered (one study noted ERT in 7/43 woman patients with +EKG/−SPECT) [3]. ERT is a well-established contributor to nonischemic ST changes during exercise stress testing [9, 10]. As chronotropic response to vasodilator stress in normal individuals is relatively brisk (particularly with regadenoson) [11], the electrocardiographic response during ERT may therefore mimic that observed during exercise.

## 2. Case Report

**Figure 1:**
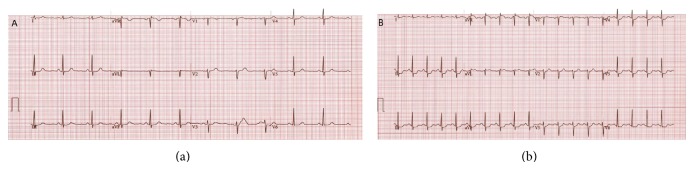
Resting (a) and stress (b) EKG tracings on patient undergoing regadenoson SPECT exam while taking chronic conjugated estrogen therapy. With pharmacologic stress, the patient developed 1.5 mm ST depression in the inferolateral distribution at a heart rate change of 50 (resting heart rate 61, peak stress heart rate 116, ΔST/ΔHR = 2).

**Figure 2:**
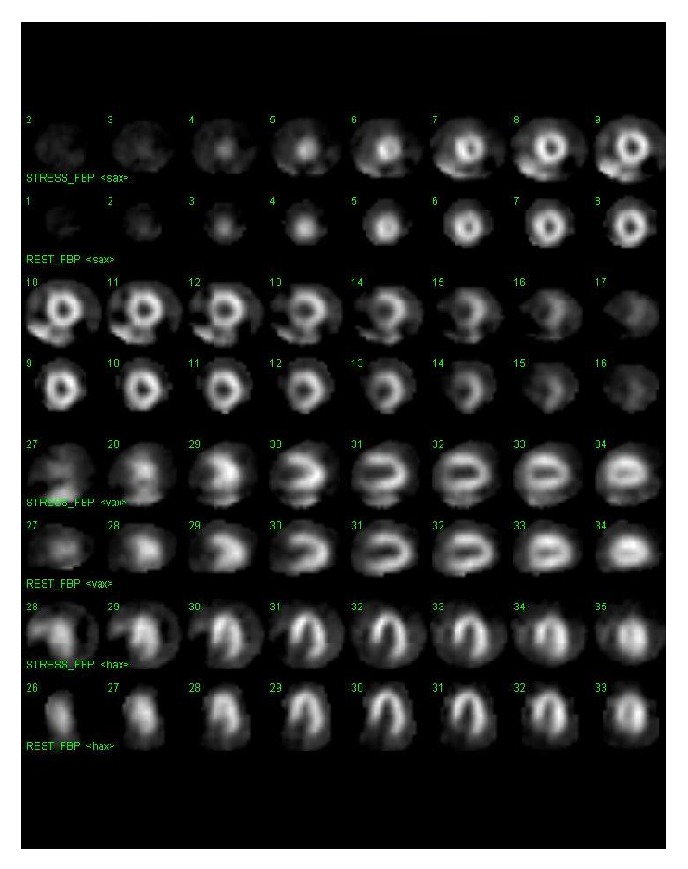
Gray scale stress (top panels) and rest (bottom panels) perfusion images, revealing absence of perfusion abnormality.

**Figure 3:**
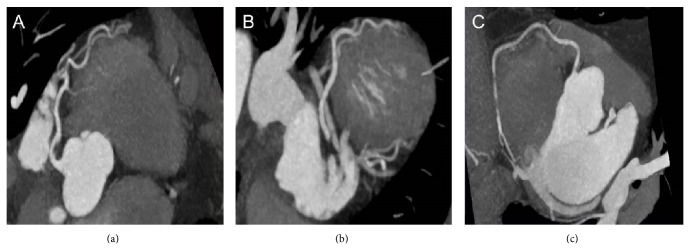
Coronary computed tomography showing absence of coronary artery calcium, with no evidence of arteriosclerotic disease.

A 60-year-old postmenopausal Caucasian woman with history of hypertension and hyperlipidemia was referred for myocardial SPECT exam with pharmacologic stress (regadenoson 0.4 mg) for evaluation of atypical angina. The patient was noted to be taking chronic ERT (conjugated estrogen 0.625 mg per day). Baseline EKG revealed normal sinus rhythm with no significant ST changes (Figure 1(a)). Following regadenoson administration, she developed 1.5 mm inferolateral ST depression (Figure 1). Stress and rest SPECT images revealed normal perfusion patterns (Figure 2), with normal left ventricular systolic function on gated images (LVEF 65%). Because of ongoing symptoms and persistent concern regarding cardiac risk, she underwent follow-up coronary computed tomography angiography with calcium scoring which revealed absence of coronary calcification or disease (Figure 3).

## 3. Discussion

To our knowledge, the occurrence of “false-positive” vasodilator EKG response associated with ERT and regadenoson is previously unreported. The case illustrates the importance of specific review and documentation of concomitant drug therapy (particularly ERT), in addition to cardiovascular risk factors when evaluating the significance of +EKG/−SPECT during pharmacologic stress. As ST depression associated with ERT use is rate-dependent, and a brisk chronotropic response to vasodilator stress (seen with regadenoson) is considered a favorable prognostic indicator [12], the latter in association with +EKG/−SPECT in patients on ERT would likely indicate a low-risk pattern.

Female-specific ischemic heart disease, erstwhile referred to as cardiac syndrome X, is associated with ST changes with exercise [13, 14]. Although this remained on the differential, our patient had pharmacologic agent-induced ECG changes, and not exercise-induced ECG changes, which made the likelihood of false-positive with ERT more likely.
